# Impact of extended [^177^Lu] Lu-PSMA-617 therapy on absorbed kidney dose and CKD-EPI values: how long can therapy be safely continued?

**DOI:** 10.1007/s00259-025-07125-1

**Published:** 2025-03-10

**Authors:** Edanur Topal, Bilal Kovan, Ayca İribas, Serkan Kuyumcu, Mert Basaran, Aydan Malçok Demirtaş, Oner Sanli, Yasemin Sanli

**Affiliations:** 1 Istanbul Faculty of Medicine, Department of Nuclear Medicine, Istanbul University, Fatih, İstanbul, 34093 Turkey; 2 Department of Radiation Oncology, Istanbul University, Istanbul University Oncology Institute, Fatih, İstanbul, 34093 Turkey; 3 Department of Medical Oncology, Istanbul University, Istanbul University Oncology Institute, Fatih, İstanbul, 34093 Turkey; 4 Istanbul Faculty of Medicine, Department of Bioistatistics, Istanbul University, Fatih, İstanbul, 34093 Turkey; 5 Istanbul Faculty of Medicine, Department of Urology, Istanbul University, Fatih, İstanbul, 34093 Turkey

**Keywords:** PSMA, Extended, Treatment, Kidney

## Abstract

**Purpose:**

This study aimed to evaluate the personalized dosimetric approach by calculating the cumulative renal absorbed dose (cRD) and assessing its impact on renal functions in patients diagnosed with metastatic castration-resistant prostate cancer who underwent four or more cycles of [^177^Lu] Lu-PSMA − 617 therapy.

**Methods:**

The study included 110 patients who received ≥ 4 cycles of [^177^Lu] Lu-PSMA − 617 therapy. Whole-body static and abdominal SPECT-CT imaging was performed at 4, 24, and 96 h post-administration. Kidney function was assessed using dynamic renal scintigraphy and biochemical tests conducted prior to treatment. Estimated glomerular filtration rate (eGFR) levels were calculated using the CKD-EPI formula before each treatment cycle and at the 6th week post-treatment.

**Results:**

Pearson correlation analysis reveal no significant relationship between cRD and CKD-EPI values (*p* >.05). No significant differences were observed between pre-treatment CKD-EPI levels and those measured after the 4th, 5th, 6th, 7th, and 8th cycles (*p* >.05). Among patients reaching a cRD of 23 Gy, a statistically significant difference was observed between pre- and post-treatment CKD-EPI values (*p* <.05). Of the 13 patients exceeding cRD of 28 Gy, five maintained CKD-EPI levels above 90 mL/min/1.73 m^2^ post-treatment.

**Conclusion:**

Despite treatment-related declines in eGFR levels, our findings indicate that a personalized dosimetric approach may enable extended cycles of [^177^Lu] Lu-PSMA-617 therapy with manageable nephrotoxicity. Considering the significant inter-patient variability, establishing universal absorbed dose-response relationships remains challenging. Prospective multicenter studies are crucial to refining toxicity thresholds and advancing tailored treatment strategies to optimize safety and efficacy.

## Introduction

Prostate cancer is a leading cause of cancer-related deaths in men [1]. Despite advancements in treatment, resistance to standard therapies such as anti-androgen regimens often necessitates alternative strategies, including radionuclide treatments like [^177^Lu] Lu-PSMA-617 [2] which has shown efficacy in metastatic castration-resistant prostate cancer (mCRPC), as demonstrated in the VISION Phase 3 trial [3]. With the consequent growing use of radionuclide therapies, dosimetric calculations have become critical to balancing tumor control and minimizing toxicity to healthy tissues [4, 5]. Dosimetric calculations in radionuclide therapies have become crucial to achieving effective treatment activities without harming critical organs.

Standardized treatment protocols often recommend 4–6 cycles of [^177^Lu] Lu-PSMA-617 based on historical data from peptide receptor radionuclide therapy (PRRT) and external beam radiotherapy (EBRT). Recent advancements in equipment and methodology, however, have enabled the integration of more precise dosimetric approaches, allowing for a more individualized application of radionuclide therapies. Numerous dosimetry studies [5–8] have demonstrated variability in absorbed doses to critical organs. These studies suggest that treatment cycles may be extended in patients who exhibit favorable therapeutic responses, as evidenced by clinical, biochemical, or imaging criteria. These findings have sparked ongoing discussions about the necessity of implementing personalized dosimetric assessments after each treatment to optimize outcomes for individual patients. In the context of [^177^Lu] Lu-PSMA therapy, organs at risk include the kidneys, bone marrow, salivary glands, and lacrimal glands. Notably, renal toxicity appears to be minimal at current activity levels, suggesting that the kidneys’ absorbed dose tolerance may exceed the doses administered thus far. This could be attributed to factors such as nonuniform radiation distribution and relatively low absorbed dose rates.

To enhance therapeutic efficacy, dosimetry-guided clinical trials have been initiated, particularly in the treatment of neuroendocrine tumors with [^177^Lu] Lu/[^90^Y] Y-PRRT. These studies often incorporate renal absorbed dose or biologically effective dose (BED) constraints, with thresholds typically set at approximately 23–28 Gy, and up to 40 Gy in patients without significant risk factors [9–11].

Building upon these insights, this study aims to evaluate the impact of personalized dosimetric approaches by calculating the cumulative renal absorbed dose (cRD) and assessing its influence on creatinine clearance levels in patients with mCRPC who have undergone four or more cycles of [^177^Lu] Lu-PSMA-617.

## Materials and methods

### Patient cohort

This retrospective study included 110 mCRPC patients treated between 2015 and 2024, all of whom had previously received anti-androgen therapy and underwent 4–12 cycles of [^177^Lu] Lu-PSMA-617. PSMA-positive metastatic foci was confirmed using baseline [^68^Ga] Ga-PSMA PET-CT imaging, with PSMA positivity defined as lesions exhibiting SUVmax values at least 1.5 times higher than the liver SUVmax [12].

Before treatment, all patients underwent baseline dynamic renal scintigraphy using [^99m^Tc] Tc-Mercaptoacetyltriglycine ([^99m^Tc] Tc-MAG-3) and biochemical tests to assess kidney function. eGFR levels were calculated using the CKD-EPI formula before each treatment cycle and at six weeks post-treatment. The formula is as follows: eGFR = 141×min (Scr/k​,1)^a^ ×max (Scr​/k,1)^-1.209^ × 0.993^age^, where k is 0.9, a is -0.411, and min and max represent the minimum and maximum values of Scr/k or 1, respectively [13].

Renal toxicity was assessed using the CTCAE v5.0 criteria, with post-treatment CKD-EPI levels categorized according to the KDIGO classification [14]. Risk factors for nephrotoxicity included retention and functional loss observed in pre-treatment dynamic renal scintigraphy, known kidney disease, comorbidities, chemotherapy, and radiotherapy history.

### Treatment protocol

**Fig. 1:**
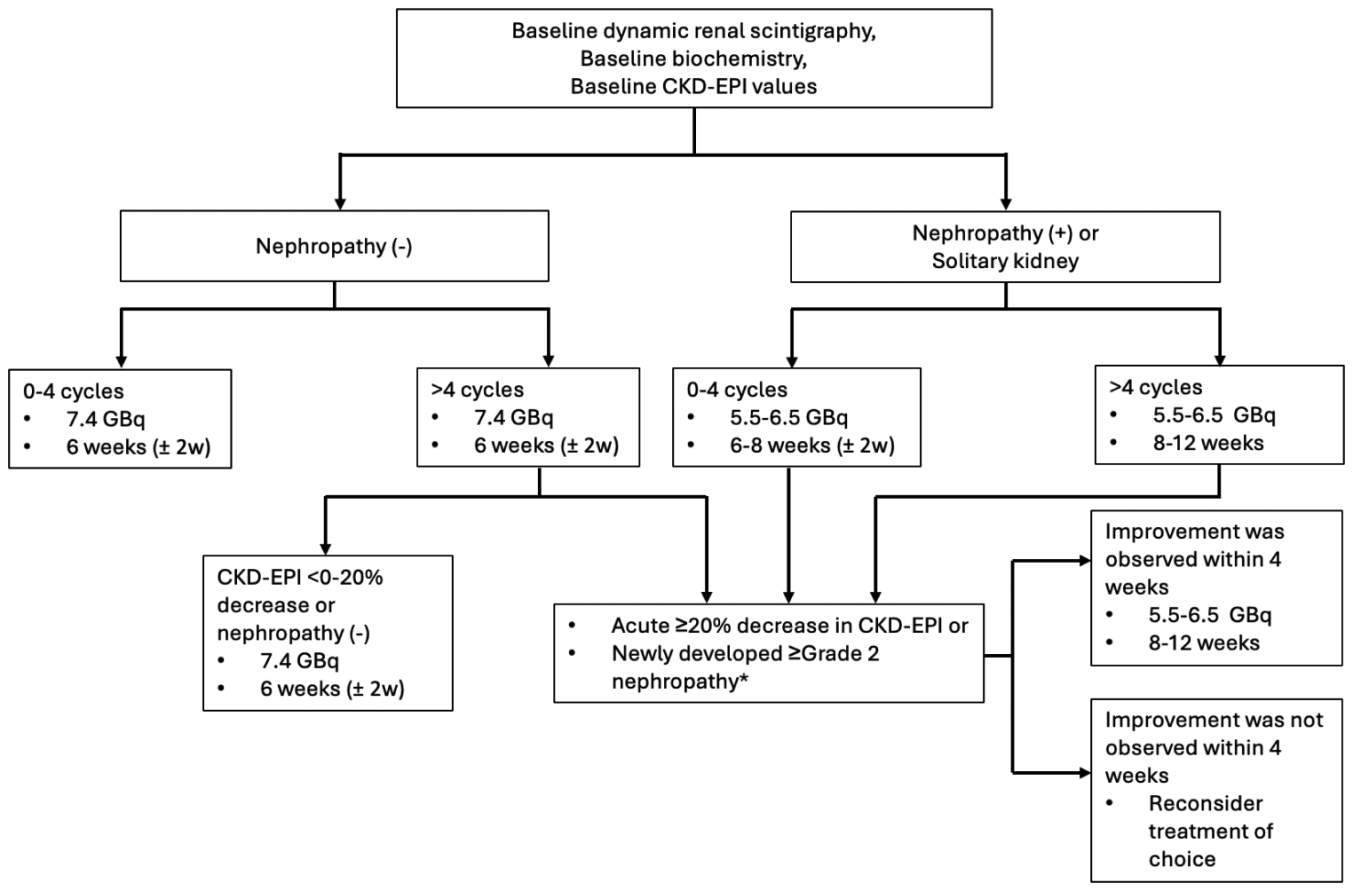
Flowchart of the treatment protocol. *According to CTCAE v5.0

Patients without nephropathy received an initial four cycles of [^177^Lu] Lu-PSMA-617 therapy, administered at 7.4 GBq per cycle with 6-week intervals (± 2 weeks). For patients with Grade 2 or higher nephropathy (KDIGO classification) or those with a solitary kidney, activity was reduced by 10% down to 5.5–6.5 GBq per cycle. Following each treatment, patients who exhibited an acute ≥ 20% decrease in CKD-EPI levels or developed Grade 2 or higher nephropathy (CTCAE v5.0) had subsequent treatments adjusted. These adjustments included reducing the activity to 5.5–6.5 GBq per cycle and extending treatment intervals to 8–12 weeks. Extended (> 4) cycles were continued if both biochemical and imaging responses were monitored and no clinical or biochemical deterioration, due to potential side effects, occurred. The treatment protocol is summarized in Fig. 1.

The decision to proceed with extended therapy was made by a multidisciplinary board. Biochemical response was assessed by a PSA decline of at least 50% 8–12 weeks after starting treatment. Interim [^68^Ga] Ga-PSMA PET scans were conducted after every two cycles of initial [^177^Lu] Lu-PSMA therapy to assess treatment response, identifying candidates for extended therapy. In addition to treatment response, special attention was given to potential side effects, including renal toxicity, hematological toxicity (e.g., anemia, leukopenia, neutropenia, thrombocytopenia), salivary gland dysfunction, and non-hematological toxicities (e.g., gastrointestinal issues, fatigue, metabolic abnormalities). If no improvement was observed within 4 weeks, treatment was temporarily paused. Severe toxicities (grade 3 or higher) were managed according to established guidelines (e.g., G-CSF for neutropenia, transfusions for anemia). Before resuming therapy, patients were reassessed for potential risks.

[^177^Lu] Lu-PSMA − 617 therapy was administered as a slow intravenous injection (3–5 min) according to the European Association of Nuclear Medicine (EANM) guidelines [10]. After each treatment, patients without cardiac conditions history received intravenous hydration with 500–1000 ml of 0.9% NaCl. Hydration was recommended in the weeks following treatment. For patients without contraindications, a single dose of 40 mg furosemide was administered 10–20 min after treatment.

### Imaging protocol

In the routine protocol, absorbed doses to the kidneys and other organs were assessed through SPECT/CT imaging conducted during the initial treatment at 4, 24 and 96 h post-administration. Imaging was performed using a dedicated SPECT/CT scanner (GE Healthcare, Discovery NM/CT 670) fitted with a Medium Energy General Purpose collimator. Whole-body (WB) images were obtained with a matrix size of 1024 × 256, scanning speed of 15 cm/min, and an energy window of 208 keV (± 10%). SPECT imaging was conducted with the patient’s abdomen and thorax region positioned within the imaging field, using 360° rotation, a 128 × 128 matrix, 60 projections, and 30 s per projection. In addition to the primary peak at 208 keV (± 10%), the scatter peak at 178 keV (± 5%) was used for scatter correction. Low-dose CT scanning was performed after the first treatment scan for attenuation correction and anatomical correlation. Image reconstruction utilized an ordered-subset expectation maximization (OSEM) algorithm with 6 iterations, 8 subsets, and no postprocessing filter. From the raw data, scattering and attenuation-corrected 3D images were generated.

### Image analysis

Image analysis was performed using OXIRIX MD software (Geneva, Switzerland). The 3D volume of interest for organs with activity was delineated from SPECT images, and organ counts were determined. For the rest of the body, counts were obtained by drawing regions of interest from the geometric means of the WB anterior and posterior images. Acquired counts were divided by the count/activity factor to calculate the [^177^Lu] Lu activities in the relevant organs and regions. These procedures were repeated for the three post-treatment images of the patient. Target organ masses were determined using CT images, and organ absorbed doses were calculated with Olinda 1.1 software.

### Statistical analysis

Statistical analysis was conducted using IBM SPSS v23.0 software. Categorical variables were presented as numbers and percentages, while continuous variables were described as mean, standard deviation, median, minimum, and maximum. Repeated measures ANOVA was used to examine changes in CKD-EPI values across absorbed doses. Mauchly’s test was conducted to assess the sphericity assumption and pairwise comparisons were performed using the Bonferroni Test to identify the source of differences between absorbed doses.

## Results

The cohort included 110 patients and patient characteristics are summarized in Table 1. Two patients with solitary kidney were included in the activity-reduced arm. In 108 patients, pre-treatment evaluations, including dynamic renal scintigraphy and biochemical tests, revealed no renal dysfunction requiring activity reduction; these patients were assigned to the standard treatment arm. In subsequent treatments, patients were reassigned to different arms based on observed side effects and cRD values. The cumulative activity of [^177^Lu] Lu-PSMA ranged from 27.0 to 83.4 GBq. The total number of treatment cycles administered across all patients was 639. In total, 4,473 images were analyzed, including 1,917 whole-body images, 1,917 SPECT images, and 639 CT images. The cRD values at the end of treatment for the ranged from 4.71 to 45.84 Gy. Considering all treatment cycles (*n* = 639), the mean absorbed dose to the kidneys per cycle was 0.46 ± 0.15 Gy/GBq. The mean cRD after 4, 6, and 8 cycles were 13.13 ± 3.63, 19.3 ± 5.11, and 27.1 ± 6.8 Gy, respectively.

**Table 1:** Patient characteristics

Parameters	
Mean age at [^177^Lu] Lu-PSMA-617 therapy	70 ± 8.4 years (range: 47–90)
Systemic therapy	
Chemotherapy	84 (76,3%)
Abiraterone/ Enzalutamide	96 (87.2%)
Median number of administered [^177^Lu] Lu-PSMA-617 cycles	6
Number of treatment cycles	(n)
4	43 (39.1%)
5	10 (9.1%)
6	19 (17.3%)
7	14 (12.7%)
8	17 (15.5%)
9	4 (3.6%)
10	1 (0.9%)
11	1 (0.9%)
12	1 (0.9%)
The mean absorbed dose to the kidneys per cycle	0.46 ± 0.15 Gy/GBq
The mean absorbed renal dose	3.3 Gy (range: 0.36–7.62)
cRD values at cycles (Gy + SD)	
4	13.13 ± 3.63
6	19.3 ± 5.11
8	27.1 ± 6.8
cRD values at the end of treatment	Mean: 19.1 ± 6.78 Gy (range: 4.71–45.84)
23 Gy	n: 12 (10.9%)
28 Gy	n: 13 (11.8%)
≥ 40 Gy	n: 3 (2,7%)
Baseline CKD-EPI levels (mL/min/1.73 m^2^+SD)	Mean: 88.13 ± 19.5
Post-treatment CKD-EPI levels (mL/min/1.73 m^2^+SD)	Mean: 82.13 ± 23.1

cRD = cumulative absorbed renal dose

**Fig. 2:**
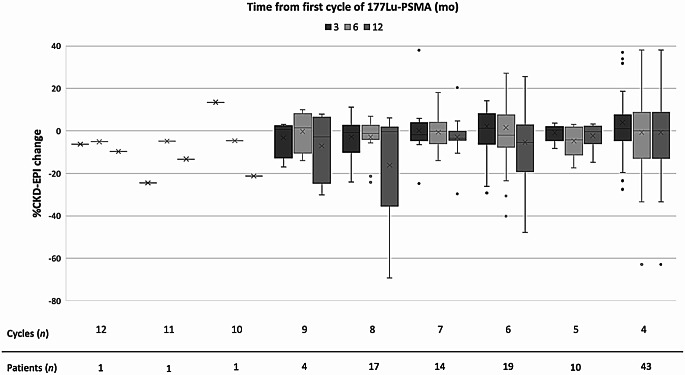
Percentage change in CKD-EPI levels over time stratified by number of [^177^Lu] Lu-PSMA therapy cycles. x within box plots = mean

Baseline CKD-EPI levels ranged from 31.0 to 113.25 mL/min/1.73 m^2^ (mean 88.13 ± 19.5). Post-treatment CKD-EPI levels ranged from 24.1 to 114.6 mL/min/1.73 m^2^ (mean 82.13 ± 23.1). Figure 2 demonstrates the distribution of patients based on CKD-EPI changes (%) following initial treatment.

**Fig. 3:**
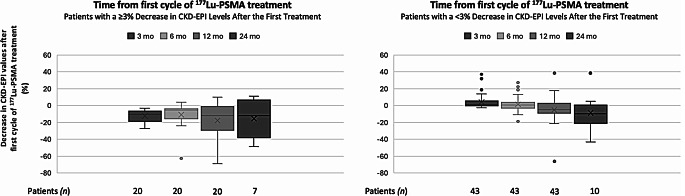
Box plots illustrating changes in % CKD-EPI over time from the initiation of [^177^Lu] Lu-PSMA treatment in 20 patients with a ≥ 3% decline at 3 months (left) and 43 patients with < 3% or no eGFR decline at 3 months (right)

Of the 110 patients included in the study, 74 (67.2%) showed a CKD-EPI decline of less than 3% at three months after the initial treatment, while 36 (32.8%) experienced a greater decline (≥3%). Among the 63 patients with a one-year follow-up, 39 from the < 3% group and 14 from the ≥ 3% group maintained a CKD-EPI decline of less than 20%. A ≥ 20% decline of CKD-EPI values was observed in 4 patients from the < 3% group and in 6 patients from the ≥ 3% group (Fig. 3). Table 2 summarizes the characteristics of the 10 patients who experienced a ≥ 20% decline in CKD-EPI levels at 12 months after initial [^177^Lu] Lu-PSMA therapy. The cohort includes patients with a mean age of 69.5 ± 5 years (range: 61–78 years), all of whom received combination treatments, including chemotherapy, radiotherapy, or hormonal therapies (Enzalutamide/Abiraterone). Notably, all patients in this group underwent chemotherapy. The number of treatment cycles ranged from 4 to 10, with renal absorbed doses varying between 2.54 and 5.11 Gy. Baseline CKD-EPI levels ranged from 64.6 to 109.8 mL/min/1.73 m^2^, with substantial declines observed at 3, 6, and 12 months post-treatment.

**Table 2:** Clinical data of patients with ≥ 20% decline in CKD-EPI levels at one-year follow-up post initial [^177^Lu] Lu-PSMA treatment

Patient Characteristics						Cycles	Activity	cRD	Mean Renal absorbed dose (Gy)		CKD-EPI values		% CKD-EPI decline after initial ^177^Lu-PSMA treatment		
Patient	Age	Ct	RT	ARPI	DRS	(n)	(GBq)	(Gy)	0–4 cycles	>4 cycles	Baseline	Post-treatment	3 mo	6 mo	12 mo
#1	69	+	+	+	N	10	70,1	44,27	4,6	4,29	81,4	65,1	13,4	-4,5	-20,3
#2	78	+	+	+	N	9	68,8	34,4	4,2	3,45	89,5	62,5	-17,0	-14,0	-30,2
#3	61	+	+	-	N	8	54,4	32,79	4,4	3,72	109,8	33,7	-9,6	-3,7	-69,2
#4	71	+	-	-	N	8	54,0	34,77	4,1	4,55	98,5	33,0	-1,4	-3,9	-66,4
#5	67	+	+	+	N	8	42,9	21,95	2,9	2,54	67,6	24,1	0	-3,9	-64,2
#6	62	+	+	+	N	8	58,8	35,13	3,8	4,9	100,0	56,8	-24,1	-24,1	-43,2
#7	70	+	+	-	N	8	54,4	23,94	3,1	2,86	99,9	72,2	-4,7	3,8	-27,7
#8	64	+	+	+	N	7	51,4	28,8	3,3	5,11	102,4	98,8	2,2	0,8	-20,3
#9	71	+	-	+	mD	6	42,2	26,91	5,16	3,13	64,6	35,0	-23,4	-23,4	-45,8
#10	72	+	-	+	N	4	27,0	14,58	3,64	-	108,3	40,3	-16,2	-62,8	-59,3

Ct = chemotherapy, RT = radiotherapy, ARPI = Androgen receptor pathway inhibitor (Enzalutamide and/or Abiraterone), DRS = dynamic renal scintigraphy, cRD = cumulative absorbed renal dose, mD = Mild delayed kidney function, mo = months

**Fig. 4:**
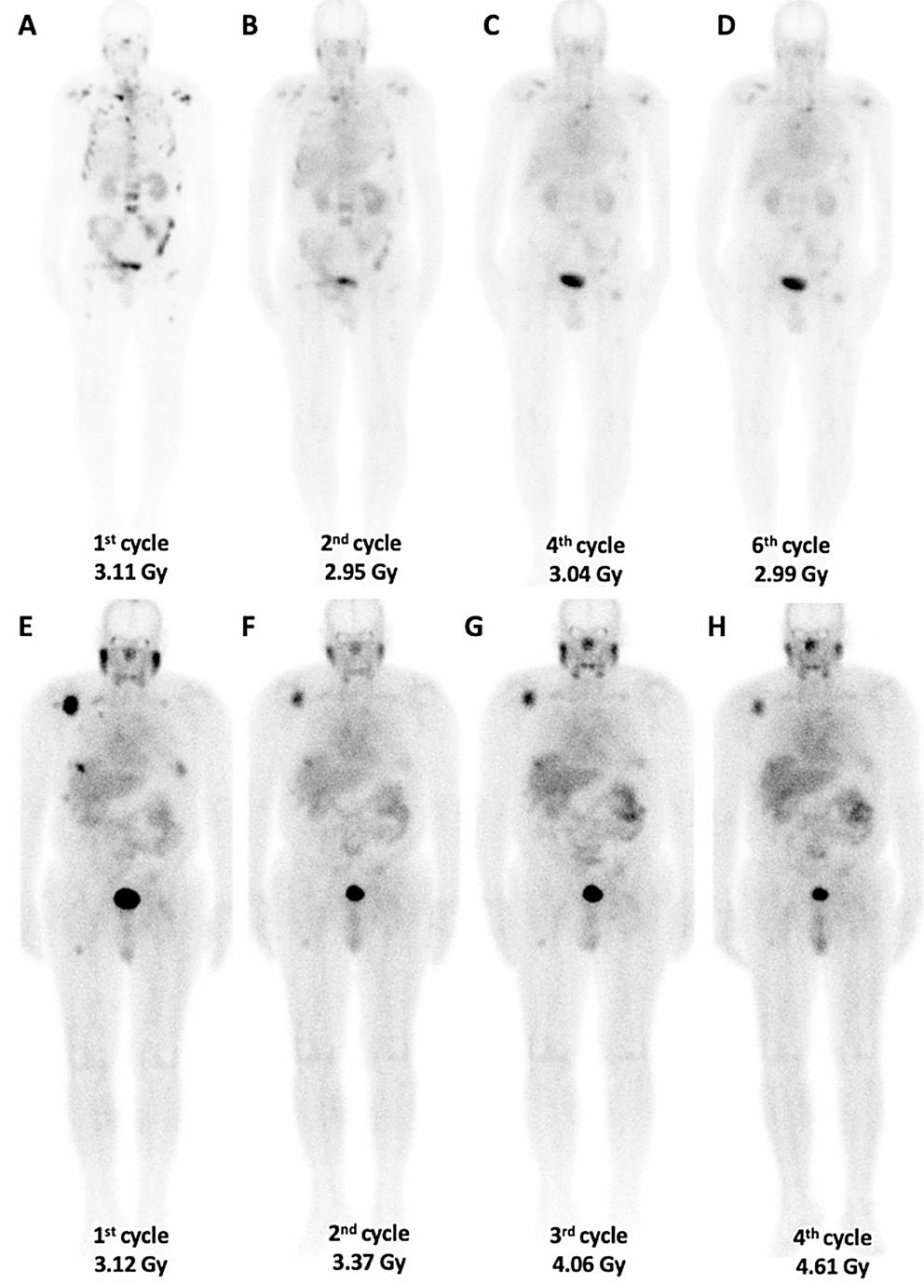
Dosimetric evaluations were conducted for mCRPC patients following treatment, with whole-body imaging performed at the 4th hour post-administration and the absorbed dose (Gy) to the kidneys during the specified treatment cycles evaluated. (*Top row*) In a 68-year-old patient with a response to treatment, including a reduction in PSA values and imaging findings, dosimetric analysis showed no significant variation in the absorbed dose to the kidneys despite a reduction in tumor burden with the same treatment activity. **A**, **B**, **C** and **D** represent images obtained after the 1st, 2nd, 4th, and 6th cycles, respectively. The calculated absorbed dose rates to the kidneys for each cycle were 3.11, 2.95, 3.04, and 2.99 Gy, respectively. CKD-EPI values for each cycle were 100.4, 106.8, 108.5, and 107.9 ml/min/1.73 m^2^. (*Bottom row*) In a 72-year-old patient showing a partial response in both PSA levels and imaging findings, dosimetric analysis revealed a gradual increase in the absorbed renal dose despite a partial reduction in tumor burden from baseline with the same treatment activity. **E**, **F**, **G**, and **H** show images from the 1st, 2nd, 3rd, and 4th cycles, respectively. The calculated absorbed dose rates to the kidneys for each cycle were 3.12, 3.37, 4.06, and 4.61 Gy, respectively. CKD-EPI values for each cycle were 99.2, 95.5, 97.5, and 95.9 ml/min/1.73 m^2^

The pre-treatment and post-treatment CKD-EPI levels and the number of patients are presented in Table 3. There was no statistically significant difference between pre-treatment CKD-EPI levels and the CKD-EPI values after the 1st, 2nd, 3rd, and 4th cycles (*p* >.05). Similarly, there was no significant difference between pre-treatment CKD-EPI levels and the CKD-EPI values after the 4th, 5th, 6th, 7th, and 8th cycles (*p* >.05). However, the differences in CKD-EPI levels between the 4th, 5th, 6th, 7th, and 8th cycles were statistically significant, as the assumption of sphericity was not met (Mauchly’s W = 0.015, *p* <.05). Therefore, repeated measures variance analysis specifically showed a significant decrease in CKD-EPI levels across these cycles (4th-8th cycles) (Greenhouse-Geisser, Huynh-Feldt, Lower-Bound, *p* <.05).

**Table 3:** CKD-EPI levels and patient numbers before and after treatment

Before treatment CKD-EPI (ml/min/1.73 m^2^)	Number of patients (*n*)	Post-treatment Patient distribution		After treatment CKD-EPI (ml/min/1.73 m^2^)	Number of patients (*n*)
		(*n*)	KDIGO groups (range of cycles)		
> 90 (G1)	65	8	G2 (4–8 cycles)	> 90 (G1)	55
		2	G3a (6–8 cycles)		
		4	G3b (4–8 cycles)		
60–89 (G2)	31	4	G1 (4–6 cycles)	60–89 (G2)	35
		3	G3a (6–8 cycles)		
		1	G3b (6 cycles)		
		1	G4 (8 cycles)		
45–59 (G3a)	8	4	G2 (4–6 cycles)	45–59 (G3a)	9
30–44 (G3b)	6	1	G2 (4–6 cycles)	30–44 (G3b)	8
		2	G4 (6 cycles)		
15–29 (G4)	0	-	-	15–29 (G4)	3
< 15 (G5)	0	-	-	< 15 (G5)	0
Total	110			Total	110

* Classified according to the KDIGO guidelines

For the 7 patients who received 9 cycles, the mean cRD was 31.75 ± 7.1 Gy. Of these, 5 had post-treatment CKD-EPI levels comparable to their baseline levels, while 2 showed a 30% decrease. Among the 3 patients who received 10 cycles, the mean cRD was 38.68 ± 5.85 Gy. One patient had post-treatment CKD-EPI level comparable to baseline, while the other 2 showed a 30% decrease. For the single patient who received 12 cycles, the cRD was 45.84 Gy (mean absorbed renal dose: 3.82 Gy ± 1.2 Gy), with a baseline CKD-EPI level decreasing from 88.5 to 54.41 mL/min/1.73 m^2^ (38% decrease).

Of the 110 patients in the cohort, 12 reached a cRD of 23 Gy, while 13 and 3 exceeded this threshold, receiving 28 Gy and 40 Gy, respectively, by the end of treatment. The mean number of treatment cycles required to reach a cRD of 23 Gy was 6 ± 1. When comparing the baseline CKD-EPI levels to those measured after reaching a cRD of 23 Gy (*n* = 27), which included patients receiving 5 cycles (*n* = 4), 6 cycles (*n* = 12), 7 cycles (*n* = 6), 8 cycles (*n* = 4), and 9 cycles (*n* = 1), a 36.2% decrease in CKD-EPI values was observed. The difference between the baseline and the 23 Gy of cRD levels was statistically significant (*p* <.05).

Among the 110 patients, 13 had a cRD of 28 Gy or greater after treatment. The mean number of cycles to reach a cRD of 28 Gy was 7 ± 0.5. Of these patients, the baseline CKD-EPI level was calculated as a mean of 97.9 ± 6.7 mL/min/1.73 m^2^, while the mean CKD-EPI levels after the treatment reaching a cRD of ≥ 28 Gy were 80.9 ± 21.5 mL/min/1.73 m^2^. Due to the small number of patients, statistical analysis could not be performed; however, a 17.4% decrease in CKD-EPI values was observed.

Of the 3 patients with a cRD exceeding 40 Gy, one had a cRD of 45.84 Gy after 12 cycles and exhibited Grade 2 nephropathy (CTCAE v5.0) (baseline to post-treatment CKD-EPI levels: 88.5–54.4 mL/min/1.73 m^2^). CKD-EPI levels of this patient improved by 10% at 3 months after the final treatment cycle and remained stable throughout the 12-month post-treatment follow-up. The remaining 2 patients had cRDs of 41.61 Gy and 44.27 Gy (patient #1) after 8 and 10 cycles, with baseline to post-treatment CKD-EPI levels; 101.8-105.5 mL/min/1.73 m^2^ and 81.4–65.1 mL/min/1.73 m^2^, respectively. Patient #1 had a CKD-EPI level of 49.5 at the end of the first year post-treatment, reflecting a 23.9% decline compared to the level at the end of treatment. However the patient had received additional chemotherapy during this period. The other patient did not have long-term follow-up data, but CKD-EPI levels remained stable at 6 weeks after last-treatment.

Two patients in the cohort has solitary kidney. The first patient had a baseline CKD-EPI level of 100 mL/min/1.73 m^2^, with a cRD of 31 Gy after 7 cycles and a post-treatment CKD-EPI level of 97 mL/min/1.73 m^2^ (3% decrease). At the 1-year follow-up, the CKD-EPI level declined to 51.62 (48% decrease). However, the patient received additional chemotherapy during the 1-year follow-up period. The second patient had a baseline CKD-EPI level of 31 mL/min/1.73 m^2^ and a cRD of 12 Gy, with post-treatment CKD-EPI levels of 27.3 mL/min/1.73 m^2^ (12% decrease). Long-term follow-up data for this patient was unavailable.

## Discussion

The [^177^Lu] Lu-PSMA-617 therapy is emerging as an effective treatment modality for mCRPC owing to its therapeutic efficacy and favorable side effect profile. However, most studies assessing its side effects have limited the treatment cycles to an empirically determined range of 4–6 cycles to avoid exceeding the absorbed dose toxicity thresholds for at-risk organs [10, 15, 16]. Toxicity limits are typically derived from the findings in radiotherapy and [^177^Lu] Lu-PRRT studies, which help define the maximum absorbed doses that can be safely absorbed by at-risk organs [17–20].

Given these absorbed dose constraints, the accurate measurement and monitoring of radiation delivery to organs are crucial. This highlights the role of dosimetry which involves key parameters such as the optimal imaging modality (planar vs. SPECT), the number of imaging sessions, and their timing. In a study on kidney dosimetry during [^177^Lu] Lu-PSMA-617 therapy, Rosar et al. found that hybrid imaging (planar and SPECT) and SPECT alone provide more accurate results than planar imaging alone [21]. The superiority of three-dimensional imaging over planar imaging lies in its ability to eliminate the excessive activity counts resulting from the overlapping of organs such as the liver and intestines, as well as metastatic tumor foci, enabling more accurate organ-based volume calculations [15, 22, 23].

Recent guidelines recommend multiple imaging sessions to determine the time-activity curve, with at least three time points: one on the treatment day (> 4 h), another at 24 h, and a final one up to 7 days later [10, 11]. In our cohort, three-time-point hybrid imaging (whole-body and SPECT) was performed at 4, 24, and 96 h after each treatment cycle, accompanied by simultaneous CT imaging for attenuation correction to calculate absorbed organ doses with high accuracy.

Given factors such as the type of radionuclide used, radiation heterogeneity, individual variations in patient physiology, the effects of cumulative absorbed doses on at-risk organs, and changes in tumor burden over the course of treatment (e.g., tumor sink effect), it is suggested that the radiobiological effect may not remain consistent across all treatment cycles (Fig. 4). The empirically established number of treatment cycles [^177^Lu] Lu-PSMA treatment based on organ toxicity limits derived from EBRT and [^177^Lu] Lu / [^90^Y] Y -PRRT, may not adequately account for the dynamic physiological and biological changes that occur during therapy. Different radionuclide therapies exhibit distinct radiobiological effects and result in varying absorbed dose distributions, highlighting the need for individualized dosimetry to optimize treatment efficacy and minimize toxicity [12, 17, 18, 24, 25]. As such, the prescribed cycle numbers might limit the potential benefits of prolonged [^177^Lu] Lu-PSMA therapy particularly for patients who have not reached toxicity thresholds in at-risk organs. Kiess et al. [26] have noted that these limitations hinder treatment optimization across various radiopharmaceuticals. For long-term toxicity-related issues, such as renal toxicity, accurately determining the absorbed dose to the affected organ is essential. In the study by Rosar et al. [27] 22 mCRPC patients who had impaired baseline kidney function (GFR ≤ 60 mL/min) received 40 GBq of ^177^Lu -PSMA therapy, and no significant decrease in GFR was observed during follow-up. Kabasakal et al. [5] calculated an average kidney dose of 0.88 ± 0.40 Gy/GBq and suggested that treatment up to 30 GBq activity could be administered without exceeding the 23 Gy kidney toxicity threshold. Kratochwil et al. [28] reported an average kidney dose of 0.75 ± 0.19 Gy/GBq and recommended that activity of 36 GBq would be appropriate for treatment. Scarpa et al. [29] found an average kidney dose of 0.60 ± 0.36 Gy/GBq and evaluated the maximum allowable activity of 61.66 ± 35.97 GBq. Our cohort study yielded an average kidney dose of 0.46 ± 0.15 Gy/GBq, consistent with findings from the VISION trial [30] (0.43 ± 0.16 Gy/GBq). Consequently, in our study, the renal absorbed dose was considered acceptable for this group of patients with limited treatment options at this stage of disease.

In our cohort, the mean number of cycles to reach a cRD of 28 Gy was calculated to be 7. For patients receiving 8 treatment cycles, the mean cRD was 27.1 Gy, remaining below 28 Gy (range: 14.99–41.61 Gy). In this group, the patient with the highest cRD of 41.6 Gy showed no decrease in CKD-EPI levels. After 9 cycles, 2 patients remained below the 28 Gy cRD. The patients who received 9 (*n* = 4), 10 (*n* = 1), 11(*n* = 1), and 12 (*n* = 1) cycles reached an average cRD of 31.7 Gy by the 9th cycle, remaining below the 40 Gy. Among the 110 patients, only 1 of the 3 patients who exceeded a cRD of 40 Gy exhibited Grade 2 nephrotoxicity (CTCAE v5.0). At the 3-month post-treatment follow-up, a 10% improvement in CKD-EPI levels was observed, which remained stable during the post-treatment 12-month follow-up evaluations. These findings underscore the complexity of absorbed dose optimization in radionuclide therapies. The renal absorbed dose or biologically effective dose (BED) limits, toxicity thresholds, highlight the need to investigate patient-specific absorbed dose limits through personalized dosimetry in [^177^Lu] Lu -PSMA therapy. Schäfer et al. [31] presented three cases of mCRPC, treated with high-activity (54.8 to 69.5 GBq) [^177^Lu] Lu-PSMA-I&T RLT, resulting in significant renal toxicity (CTCAE grade 3–4 chronic kidney disease, CKD). The authors reported the patients underwent 8–10 treatment cycles, CKD onset occurred 12–17 months after the first cycle and continued to worsen even after treatment cessation. Despite the substantial treatment, most patients did not experience significant renal damage, highlighting the variability in renal toxicity.

A decline of more than 20% per year in creatinine clearance over a consistent annual period has been used as an indicator of end-stage renal disease [32]. In our study, among 63 patients with 1-year follow-up data after the first treatment, 10 patients exhibited a ≥ 20% decline in CKD-EPI levels (Table 2). In this group, 6 out of 10 patients showed a ≥ 3% decline in CKD-EPI levels after 3 months of the first treatment. Due to the limited number of patients, statistical evaluation could not be performed; however, in patients with a ≥ 3% decrease in eGFR at 3 months after treatment initiation, more progressive rates of decline were observed at the 6-month and 12-month follow-up compared to the group with a < 3% decline (Fig. 3). Nevertheless, it should be noted that among the 20 patients with a ≥ 3% decrease, only 6 showed a ≥ 20% decrease at the 12-month follow-up (Table 2 patients marked with # 2, 3, 6, 7, 9, and 10). These 6 patients cRD ranged between 17.7 and 42.2 Gy. This is consistent with the findings of Steinhelfer et al. [33], where patients with a ≥ 3% decrease in eGFR during the early period (3 months) showed continuous decline without stabilization or recovery throughout follow-up. These results highlight the potential long-term renal risks for patients experiencing early declines in eGFR, suggesting the need for further monitoring and risk assessment in similar treatment protocols.

Establishing treatment-specific absorbed dose-response relationships and defining standard cycle numbers or activities applicable to all patients is extremely challenging. In addition to the intensive treatments patients have previously undergone, the absorbed dose rates in at-risk organs vary across standard treatment protocols. The variability among patients is undeniable and is expected to persist and intensify throughout ongoing treatments. Furthermore, it must be acknowledged that numerous factors will exert different effects at each stage of the process, potentially influencing outcome. Therefore, establishing generalized absorbed dose limits for renal toxicity may not be feasible. This highlights the necessity for personalized dosimetry and individualized patient assessments to ensure optimal treatment outcomes.

## Limitations

The limitations of our study include a small number of patients with a cRD of 40 Gy or higher, limited follow-up data over a short duration, and a relatively small sample size, which restricts the generalizability of the findings. Additionally, the single-center design limits applicability to other healthcare settings, and the short follow-up period hinders the evaluation of long-term kidney function changes and the durability of therapeutic effects.

## Conclusion

Despite a decline in eGFR levels, a personalized dosimetric approach could enable extended cycles of [^177^Lu] Lu-PSMA-617 treatment without severe toxicity. Assessing cRD levels proved crucial for evaluating the feasibility of prolonged treatments in responsive patients without significant nephrotoxicity. Further prospective, multicenter studies are essential to overcome the challenges of establishing universal absorbed dose-response relationships for treatment-specific toxicity as they would provide robust data to refine toxicity limits and support the development of tailored treatment approaches, ensuring both safety and efficacy in diverse patient populations.
